# Microemulsion Derived Titania Nanospheres: An Improved Pt Supported Catalyst for Glycerol Aqueous Phase Reforming

**DOI:** 10.3390/nano11051175

**Published:** 2021-04-29

**Authors:** Andrea Fasolini, Erica Lombardi, Tommaso Tabanelli, Francesco Basile

**Affiliations:** Dipartimento di Chimica Industriale “Toso Montanari”, Alma Mater Studiorum-Università di Bologna, Viale del Risorgimento 4, 40136 Bologna, Italy; andrea.fasolini2@unibo.it (A.F.); erica.lombardi3@gmail.com (E.L.); tommaso.tabanelli@unibo.it (T.T.)

**Keywords:** microemulsion, titania nanospheres, aqueous phase reforming, hydrogen production, weak acidity, Pt/TiO_2_

## Abstract

Glycerol aqueous phase reforming (APR) produces hydrogen and interesting compounds at relatively mild temperatures. Among APR catalysts investigated in literature, little attention has been given to Pt supported on TiO_2_. Therefore, herein we propose an innovative titania support which can be obtained through an optimized microemulsion technique. This procedure provided high surface area titania nanospheres, with a peculiar high density of weak acidic sites. The material was tested in the catalytic glycerol APR after Pt deposition. A mechanism hypothesis was drawn, which evidenced the pathways giving the main products. When compared with a commercial TiO_2_ support, the synthetized titania provided higher hydrogen selectivity and glycerol conversion thanks to improved catalytic activity and ability to prompt consecutive dehydrogenation reactions. This was correlated to an enhanced cooperation between Pt nanoparticles and the acid sites of the support.

## 1. Introduction

The aqueous phase reforming reaction (APR) of oxygenated hydrocarbons was firstly introduced in 2002 by Dumesic et al. [1] This approach represents an interesting step for the upgrading of sugars and polyols towards the production of hydrogen and liquid products in water at relatively low temperatures (150–250 °C) and pressures (15–60 bar) [2,3,4]. Among the use of ethylene glycol [3,5,6,7,8], glucose [9,10,11,12], and biomass-derived polyols [11,13,14,15,16,17,18,19] as starting raw materials, glycerol represents an interesting substrate due to its availability as coproduct of biodiesel manufactures. In this context, glycerol conversion to high added value products is considered a key factor to boost the economic viability of biodiesel production. Noteworthy, for the great majority of the proposed valorization pathways, glycerol needs to be refined and purified, increasing the cost of the overall process [20,21]. Therefore, APR allows to valorize this raw material starting from the crude diluted glycerol aqueous solution, providing consistent hydrogen production together with the formation of interesting liquid products [22,23,24]. Hydrogen can be used as a green energy carrier and fuel, thanks to its ease of stock and transport and its energy efficient conversion to electricity through fuel cells. In addition, platform molecules such as lactic acid, 1,2-propanediol, and propionic acid are co-produced in liquid phase [3,8,14,25,26,27,28]. Glycerol aqueous phase reforming (APR) is an endothermic reaction (ΔH^0^_25 °C_ = 128 kJ/mol), for this reason the majority of the scientific literature are focussed in the range 200–250 °C in order to overcome thermodynamic limitations and foster the kinetics [3,8,14,26]. On the other hand, higher temperatures are not commonly investigated due to both glycerol relatively low boiling point and to the possibility of degradation reactions to oligomers and carbonaceous species.

**Table 1:** Reactions involved in the process and associated reaction enthalpy at 25 °C.

Reaction		ΔH^0^_25 °C_
Glycerol Aqueous Phase Reforming (APR)	C_3_H_5_(OH)_3_ (l) + 3H_2_O (g) → 3CO_2_ (g) + 7H_2_ (g)	128 kJ/mol
Water Gas Shift (WGS)	H_2_O + CO → H_2_ + CO_2_	−41 kJ/mol
CO methanation	3H_2_ + CO → CH_4_ + H_2_O	−206 kJ/mol
CO_2_ methanation	4H_2_ + CO_2_ → CH_4_ + 2H_2_O	−165 kJ/mol

Together with the overall APR reaction (Table 1), several liquid phase processes need to be considered, leading to a complex pathway of interconnected reactions. In particular, hydrogenation, dehydrogenation, condensation, hydration, and dehydration mechanisms occur starting from both glycerol and reaction intermediates [29,30,31]. Other reactions that may occur to some extent in the APR process are water gas shift (WGS) and methanation reactions from CO and CO_2_ that may allow the full consumption of CO and the production of methane (Table 1) [30,32]. It is worth noting that water gas shift fosters hydrogen production, while methanation consumes the desired product and is thus to be avoided when H_2_ formation is the goal. On the other hand, the production of alkanes via Fischer-Tropsch is usually negligible in the reported conditions, thus alkanes are supposed being produced from secondary reactions in liquid phase and not from CO_x_ and H_2_ [33]. Given the complexity of this liquid/gas phase system, the main outcome may be the possibility of driving the reaction toward selected liquid or gas phase products by the careful design of the catalyst properties [3,34]. Among different supported noble metals catalysts, the selectivity in hydrogen production is reported as maxima for Pt, followed by Ni, Ru, Rh, and Pd [3]. Pt couples high C–C cleavage and WGS activity together with low C–O cleavage, favoring hydrogen formation over alkanes. On the other hand, different catalytically active supports have been investigated and proved to drive the catalyst activity and selectivity. Pt/Al_2_O_3_ was the most investigated catalyst, providing tunable morphology and wide availability [5,29,35,36,37,38]. More recently, Pt supported over such MgO, CeO_2_/ZrO_2_, and TiO_2_ have been reported [26,39,40,41,42,43,44,45,46]. MgO provided sites that allowed base catalyzed reactions, while Ce/Zr oxides gave redox sites thanks to the switch between the Ce^3+^/Ce^4+^ couple [26]. TiO_2_ has rarely been considered as support for glycerol APR [40,45]. However, Pt/TiO_2_ is reported to provide the highest hydrogen production rate for ethylene glycol at 225 °C with higher values than Pt-black, Pt/carbon, Pt/Al_2_O_3_, and Pt/ZrO_2_ [47]. For these reasons, Pt/TiO_2_ can be selected as a case study to produce liquid or gas phase products in glycerol APR. Moreover, TiO_2_ surface characteristics can be tuned through the preparation method, to produce suitable properties for APR, such as high surface area, high Pt dispersion, and high density of acid sites of suitable strength. This makes TiO_2_ a suitable material for different applications. For instance, it is widely used as electrochemical catalytic support for electro-oxidization in fuel cells [48,49,50]. In addition, it has been widely applied as a photocatalyst in wastewater treatment [51,52], pollutants abatement [53,54], solar hydrogen production [55,56,57,58], and oxidation reactions [59,60]. In general, the modification of TiO_2_ properties is a focal point of current research [61,62]. In the field of nanomaterials, the use of microemulsion approach is widely adopted in the synthesis of metal nanoparticles [63,64,65,66] and their alloys, but several studies have been also reported for the synthesis of oxides [67,68,69,70,71,72,73,74,75]. The synthesis of inorganic oxides consists in the formation of an inverse microemulsion, also called water-in-oil microemulsion. It consists in a thermodynamically stable system created when an organic solvent is mixed with an aqueous solution in the presence of one or more surfactants and characterized by the presence of water micelles dispersed inside the organic phase. These micelles can be used as confined reaction environment, where the precipitation of the desired oxide occurs in a confined and controlled environment, leading to the formation of nanospheres [70,73]. Up to date, the microemulsions synthesis of TiO_2_ is mainly followed by a thermal treatment at 120 °C, which helps to recover the produced solid, but increases crystal size and lowers surface area. Therefore, the present study is devoted to enhancing TiO_2_ surface area and properties modifying a water-in-oil microemulsion synthetic technique, to produce small and narrow distributed nanocrystals with high surface area and suitable properties for the desired reaction. The obtained material was impregnated with Pt and applied to glycerol APR. The results outperformed those obtained with a commercial TiO_2_ support. Finally, a mechanism for glycerol APR was proposed following product formation at different reaction times and with different intermediates.

## 2. Materials and Methods

### 2.1. Synthesis of TiO_2_ by Microemulsion (TiO_2_-m)

#### 2.1.1. Synthesis Background and Current Modifications

TiO_2_-m was synthesized by water-in-oil microemulsion [73,74,76]. In a microemulsion, TiO_2_ hydrolysis occurs between Ti organic precursor (TBT) and water at the interface between aqueous and organic phases [73,77]. The presence of an organic phase also favors the solubility of the organic Ti precursor compared to aqueous solutions as well as the solubility of the hydrolysis byproduct, i.e., 1-butanol [73]. The TBT molecules solved in the organic phase migrate toward the micelles where they get in contact with the aqueous environment thanks to the flexibility of the micelle shell. There hydrolysis by water insertion occurs, while the leaving 1-butanol by-product is removed from the aqueous environment due to its higher affinity with the organic phase, driving the reaction toward product formation.

As the reaction is influenced by the micelle shell dimension, this technique allows to control TiO_2_ nanoparticles formation and growth. In particular, smaller micelles giving smaller nanospheres [73]. In literature studies, TBT solution is usually added dropwise to a water-in-oil microemulsion [73,74,78,79,80], which, however, may alter the micelle dimension leading to bigger nanospheres. The formation of TiO_2_ particles is usually followed by a hydrothermal treatment in autoclave at 120 °C [78,79,80,81,82], this step is used to break the micelles of the microemulsion, which allows the recovery of the solid and increases the material crystallinity, particle size, while, however, decreasing surface area. The higher the hydrothermal treatment temperature, the higher is the increase in particle size and decrease in surface area [79]. Although this finding has specific applications, it is opposing to the aim of this study, where small and high surface area nanospheres are desired to boost the APR reaction. For this reason, the addition mode and thermal treatment of already reported microemulsion were modified to minimize particle dimension and maximize surface area. A water in heptane microemulsion was formed to which a solution containing the Ti precursor (TBT), heptane and the surfactants was added. The presence of the solvent and of the surfactants in the TBT allowed to keep small micelle dimension, controlling the hydrolysis and condensation to TiO_2_. Moreover, the hydrothermal treatment step was substituted by heating the microemulsion to reflux (74 °C). This allowed to break the micelles and recover the solid titania, without drastically increasing the particle size and decreasing the surface area.

#### 2.1.2. Synthetic Procedure

Cyclohexane (99%, Sigma-Aldrich, Milan, Italy) was used as oil phase, Triton X-100 (Sigma-Aldrich, Milan, Italy) was used as surfactant and 1-hexanol (98%, Sigma-Aldrich, Milan, Italy) as co-surfactant. The ratio between oil and surfactant was 1.17 (*wt/wt*) and that between co-surfactant and surfactant was 0.46 (*wt/wt*). A first microemulsion (solution “A”) was synthesized by slowly adding under stirring a 5 M HNO_3_ (68%, VWR, 10.3 mL) solution in distilled water to the organic components (hexanol 3.1 g, TX-100 6.9 g, and cyclohexane 8.0 g). The organic titanium precursor (TBT) (97%, Titanium tert-butoxide, Sigma-Aldrich, 4.4 g) was added to a second solution of organics (hexanol 3.1 g, TX-100 6.9 g, and cyclohexane 8.0 g; solution “B”). Then, “B” was slowly poured in “A” under vigorous stirring. The hydrolysis of the precursor inside the micelles to form the oxide was allowed to occur by stirring at room temperature for 1, 24, or 48 h. Subsequently, the microemulsion was brought to reflux temperature (74 °C) and kept for 5 h.

To synthesize TiO_2_-5 days, the microemulsion obtained after the mixing of “A” and “B” was stirred for 5 days at room temperature with no further heating. To synthesize TiO_2_-120 °C, the microemulsion obtained after the mixing of “A” and “B” was stirred at room temperature for 1 h, then poured into an autoclave stirred at 120 °C under autogenous pressure for 4 h.

The solid recovery was accomplished by centrifugation and washed 5 times with ethanol. After drying at 120 °C overnight, the powder was calcined at 400 °C for 3 h (ramp 2 °C/min). Although calcination decreased the surface area of the support (as reported in the results section), it was carried out to provide a material which were stable under APR conditions. The support obtained in this way was named as TiO_2_-m400.

### 2.2. Impregnation of the Active Phase

TiO_2_-m and commercial TiO_2_-comm (DT-51 CrystalACTIV™) were loaded with Pt 1% or 3% *wt/wt* of catalyst, by Incipient Wetness Impregnation of tetraamine platinum (II) nitrate (99.99%, Alfa Aesar, Premion^®^) in distilled water. The powders were put in an oven 120 °C, then calcined heated up at 350 °C with a ramp of for 2 °C/min and kept for 3 h. Reduction to Pt^0^ phase was fulfilled heating under a 10 mol% H_2_ in nitrogen flow (100 mL/min) at 350 °C for 3 h.

### 2.3. Characterization of the Catalyst

Dynamic light scattering (DLS, Malvern ZetaSizer, Rome, Italy) was used to analyze microemulsions using quartz cuvette. The hydrodynamic diameter valuated the coordination sphere and the adsorbed species such as surfactants and cosurfactant. Scanning electron microscopy/energy dispersive spectroscopy (SEM/EDS) analyses were carried out using an E-SEM Zeiss EVO 50 Series Instrument (Carl Zeiss s.p.a. Milan, Italy) equipped with an INCA Energy 350 EDS micro analysis system (Oxford Instruments Analytical, Abingdon, UK). The accelerating voltage was 20 kV and the spectra collection time 60 s. Philips X’Pert X’Celerator, with Cu-k_α_ radiation in the range 5–80°2θ with step of 0.1°2θ was used to characterize the powders by X-ray diffraction (XRD). Scherrer equation was used to calculate average crystallite size, which was approximated to average particle size for the purpose of this work. Anatase/Rutile phase ratio was obtained by Rietveld refinement. ASAP 2020 (Micromeritics instrument, Norcross, GA, USA) was used to analyze the powders using N_2_ as probe gas. Ammonia temperature programmed desorption analyses were carried out in a Autochem II instrument (Micromeritics instrument, Norcross, GA, USA). The sample (0.150 g) was first pretreated at 400 °C for 45 min, then cooled to 100 °C in inert atmosphere. It was flushed for 1 h with a 10 mol% of NH_3_ in He (30 cm^3^/min) at 100 °C, then it was purged for 1 h in helium. Finally, a ramp of 10 °C/min to 650 °C and held for 30 min was used for the desorption measurements. Impregnated catalysts were analyzed by High Resolution Transmission Electron Spectroscopy (HR-TEM) with Transmission Electron Spectroscopy/Scanning Transmission Electron Spectroscopy TEM/STEM (FEI Tecnai F20), which a High-Angle Annular Dark Field (HAADF) imaging mode at 200 kV. Microwave plasma atomic emission spectroscopy (MP-AES) experiments were carried out on the liquid product solution after filtration of the solid catalyst using an 4210 MP-AES (Agilent Technologies, Milan, Italy).

### 2.4. APR Reaction

A 300 mL stainless steel Parr autoclave was used to perform APR test and loaded with a 17 wt% (or 6 wt%) solution of glycerol in water and 0.45 g of catalyst. Different amounts of glycerol were used to investigate their effect on conversion and carbon loss. In general, higher carbon losses were obtained at higher concentrations, but in this case, the difference in the formation of liquid products was higher helping the comprehension of the reaction mechanism involved. The system was flown with purged under N_2_ before the test to avoid oxygen presence, then heated to the desired temperature with a rate of 4.2 °C/min, starting from atmospheric pressure. For simplicity, the reaction time reported in the results does not consider the heating period. All the reactions were performed between 200 and 250 °C, at autogenous pressure for different reaction times. Reactions conducted for 0 h indicated that the autoclave was heated up to the desired temperature and immediately cooled down to room temperature. Working in autogenous conditions allowed to keep the system in an equilibrium between gas and liquid phase. Gas analyses were carried out off-line in a Thermo Focus GC with a CARBOSPHERE 80/100 6 × 1/8 column and a Thermal Conductivity Detector (TCD detector). Liquid products were analyzed with an Agilent HPLC over Rezex ROA Organic Acid column (0.0025 M H_2_SO_4_ mobile phase at 30 or 60 °C and a flux of 0.6 mL/min) with Diode-Array Detector (DAD) and Refractive Index Detector (RID) detectors.

The mean of five injections were used to calculate gas products selectivities. Stoichiometric factor (*ν*) was calculated considering the stoichiometry of the transformation of a glycerol molecule into the selected product *i*:

$$S_{i}=\frac{n_{i\;inj}\;}{n_{inj\;tot}}\times \frac{P_{syst}\times V_{syst}}{R\times T\left(K\right)}\times \frac{\nu \times 100}{\left(n_{0\;gly}-Conc._{gly}\times V{\left(L\right)}_{liq}\right)},$$

For instance, *ν* = 1/4 for H_2_; 2/3 for C_2_H_4_, C_2_H_6_; 1/3 for CO, CO_2_, CH_4_; 1 for C_3_H_8_.

Liquid product selectivities were calculated as follows. Stoichiometric factor (*ν*) was calculated, taking in account the stoichiometry of the reaction of a glycerol molecule into the selected product *i*:

$$S_{X}=\frac{n._{i\;}}{\left(n_{\;gly\;conv.}\right)}\times \nu \times 100,$$

*ν* = 1 for C_3_ products; 2/3 for C_2_ products. Products with low selectivities were identified but not quantified.

## 3. Results

### 3.1. Optimization of the TiO_2_ Microemulsion Preparation

**Figure 1:**
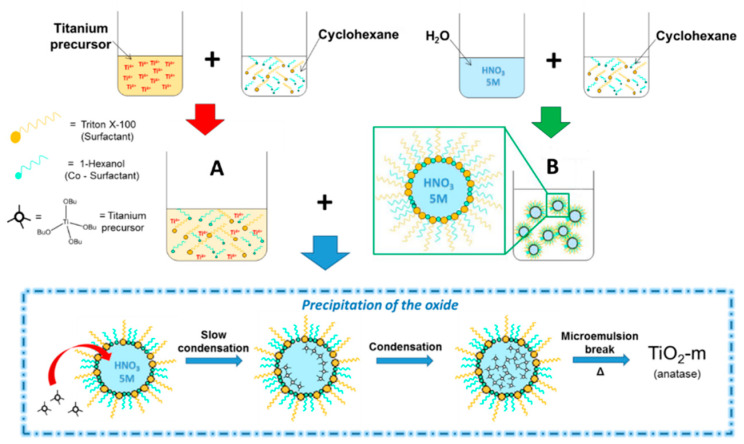
Schematization of the modified microemulsion preparation procedure.

Microemulsion based synthesis has been used in literature to obtain TiO_2_. However, relatively low surface area materials were obtained, due to the employment of hydrothermal treatments at 120 °C. The objective of this work was to modify the synthesis to obtain high surface area, small and regular TiO_2_ nanospheres, suitable for the APR reaction. To fulfill this aim, a novel synthetic procedure was developed, starting from already reported syntheses [73,74,76], modifying the addition method and the thermal treatment steps. In particular, the organic Ti precursor (Titanium tert-butoxide, TBT) was dissolved in the same organic components (heptane, 1-hexanol, and Triton X-100) which are present in the microemulsion system (Figure 1). In this way, when the Ti-containing solution was added to the microemulsion, the presence of heptane and surfactants helped to preserve small micelles which favor the formation of small TiO_2_ particles as their dimension is correlated to the dimension of the crystallized solid [66]. Moreover, hydrothermal heating at 120 °C was substituted by a heating step to reflux (74 °C). This allowed to break the micelles and recover the solid (Figure S1), without favoring particle enlargement.

**Figure 2:**
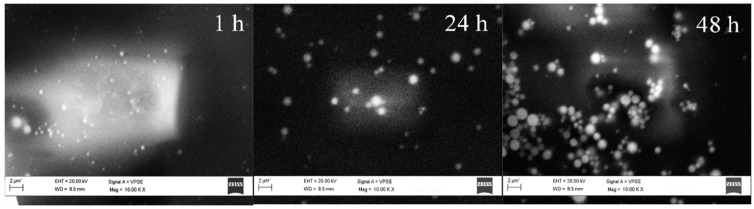
SEM images of the powder obtained by microemulsion after 1, 24, and 48 h of stirring at room temperatures. Volatile compounds were removed by drying at 120 °C.

**Figure 3:**
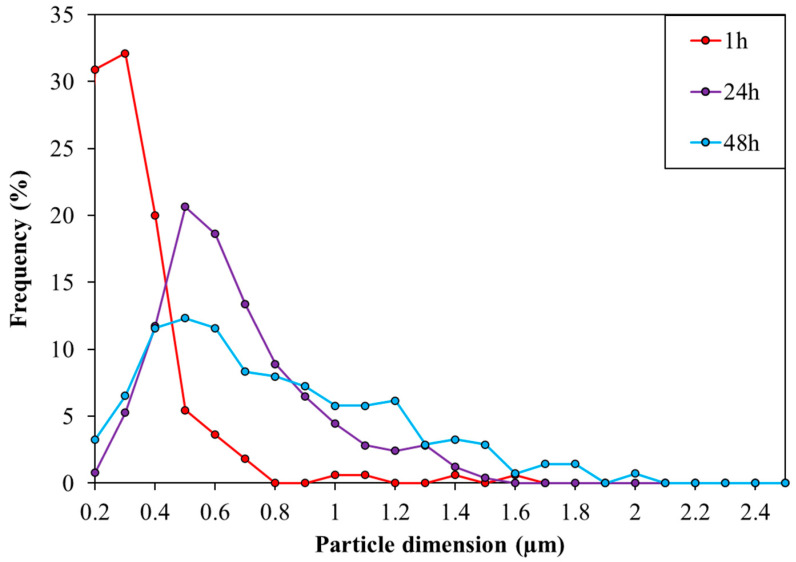
Distribution of TiO_2_ particle dimension obtained with Scanning Electron Microscopy (SEM) analyses as a function of the different stirring times. 0 h: no particles can be detected using SEM.

To further control the dimension of the TiO_2_ particles, the effect of reaction time after the mixing of the two microemulsions was investigated. Different reaction times, namely 0 h (just mixing the reagents), 1, 24, or 48 h were screened. Particular attention was given to the formation of precipitate, following the microemulsion micelle dimension by dynamic light scattering (DLS) and characterizing the produced solid, dried at 120 °C, using the SEM (Figure 2 and Figure 3). DLS showed micelles with a mean diameter of 6 nm at the beginning of the reaction (0 h), while after 1 h of stirring micelles with a 6 nm diameter were still present together with larger species around 4–5 µm, indicative of particle coalescence and the presence of a precipitate. The size distribution obtained by SEM described an increase in particle size with stirring time. No particle was observed after 0 h, probably due to uncomplete hydrolysis or the presence of particles smaller than 0.2 μm which is the sensitiveness limit of the instrument. A clear particle growth was observed increasing the stirring time with particles of 0.2–0.3 μm after 1 h, followed by a broader size distribution at 24 h, centered at 0.5 μm. Finally, a multimodal particle size distribution with particles in the range of 0.8–1.8 μm was observed after 48 h.

**Figure 4:**
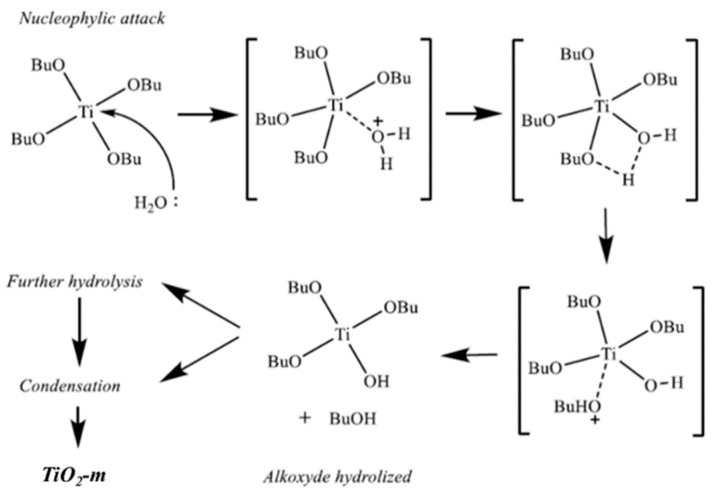
Mechanism of titania formation from TBT in water environment. Adapted from [73].

The obtained particle distribution suggests that the formation of TiO_2_ from an organic precursor occurs by a hydrolysis and condensation mechanism (Figure 4). TBT hydrolysis occurs when a (i) water molecule gives a nucleophilic attack on Ti, (ii) a transition state with a fivefold coordinated Ti is formed, and (iii) water transfers a proton to the alkoxide group that leaves in the form of an alcohol. This mechanism can be followed by two pathways, namely further hydrolysis on the same precursor molecule or condensation between hydrolyzed molecules forming Ti-O-Ti bonds.

In aqueous solutions, titanium tert-butoxide hydrolysis is dependent on the acid concentration. When TBT was added to a neutral or basic solution, the formation of a white solid was observed. The same phenomenon was observed in 0.5 M HNO_3_, while if the acid concentration was increased to 5 M, the TBT-water solutions was stable for several days, avoiding the formation of a solid. For this reason, 5 M HNO_3_ was selected as the microemulsion aqueous phase, being able to slow down the hydrolysis process, allowing a better control on the desired phenomena. In fact, the presence of the acid and of the interface, allows a slow hydrolysis to happen, making the overall process controlled by condensation. During the first stages of the synthesis, condensation occurs to a low extent, forming a small particle. As the reaction time increases, condensation leads to particle enlargement, as detected by SEM.

Thus, the synthesis with 1 h aging time was selected as the most promising for application in glycerol APR, as smaller TiO_2_ particle size was shown to increase metal dispersion and reforming activity [83].

For a deeper insight into the role of the microemulsion system as a reaction confinement environment, another synthesis was carried out through the same procedure, though in the absence of surfactant and co-surfactant, forming of an unstable biphasic system which produced an emulsion when stirred. The microemulsion synthesis developed in this work was characterized by a mainly anatase phase (93 wt% with a 7 wt% of rutile, as determined by Rietveld refinement) with small particles of an average size of 4 nm and a high surface area of 319 m^2^/g (Table 2 and Figure S2). On the other hand, in the absence of surfactants, a high rutile fraction (64%) was observed, with small particles (5 nm), but lower surface area (199 m^2^/g) due to the absence of a controlled hydrolysis. Thus, the presence of small and thermodynamically stable micelles dramatically affects the synthesis output as they act as a confined reaction environment providing a more controlled environment, which slows down the hydrolysis rate and provides higher surface areas and a complete shift from the mostly-anatase phase.

Some other parameters were studied, analyzing the effect of longer stirring times or the effect of the addition of a hydrothermal treatment performed in autoclave. One sample was stirred at room temperature for 5 days with no further heating, while another one was stirred at room temperature for 1 h and then treated at 120 °C in hydrothermal conditions for 4 h. The samples were characterized by BET and XRD analyses and average crystallite size was calculated with the Scherrer equation from the X-ray diffractogram (Table 2, Figure S3). When stirred for 5 days with no further heating, the microemulsion synthesis led to the formation of a mainly rutile phase, with larger crystallites and lower surface area, as previously reported [73]. Finally, the hydrothermal treatment (TiO_2_-120 °C) provided a sample with a surface area of 249 m^2^/g and a pure anatase phase. Thus, the employment of short reaction times (1 h) and reflux heating instead of hydrothermal treatment in autoclave allowed to increase the surface area of the material to 319 m^2^/g, almost three times higher than that obtained with 5 days stirring and still much higher that the hydrothermal treated sample.

It is interesting to discuss the formation of anatase and rutile depending on the synthetic conditions. As discussed previously, the formation of TiO_2_ occurs through hydrolysis and condensation steps. When TBT reacts with H_2_O present in the micelles, the coordination of the titanium ion is increased when an electron pair of the oxygen is accepted by a vacant d orbital. This leads to an octahedral structure of the type Ti(O)_m_(OH)_n_(H2O)_6−m_^(2m+n−4)^ [84]. At this stage, dehydration occurs and condensation starts. Here, the formation of TiO_2_ crystals involves TiO_6_^2−^ octahedra which shares edges and corners in different configurations leading to different TiO_2_ polymorphs [85]. If four edges are shared, the crystal grows towards the (221) miller index and results in the formation of anatase, in what is called zig-zag packing [86]. On the opposite, rutile is formed when the octahedra share two edges to form the (001) plane, in a linear packing. A good graphical representation of this process has been reported in literature [85,86]. A substantial difference in the formation of rutile or anatase relies in the coordination of TiO_6_^2−^ anions: *cis*-coordination sites are involved in the case of anatase formation while rutile requires a *trans*-coordination to occur [86]. Although the *trans*-coordination is the thermodynamically stable one, it is unstable under kinetically controlled conditions [86].

Following the experimental results herein reported it can be stated that rutile is more easily formed at longer reaction times (TiO_2_-5 days) or in larger micelles, as proved by the phase composition of TiO_2_-e, where the absence of surfactants gave an emulsion with larger water droplets. Both these situations led to a thermodynamic product as TiO_6_^2−^ anions are freer to rearrange, due to a higher reaction time or larger droplets. On the opposite, if the microemulsion was stirred for 1 h, the constrained aqueous environment and the low reaction time led to the formation of the kinetic product, namely the metastable anatase (Figure S4).

Given its higher surface area the support stirred for 1 h in a microemulsion (TiO_2_-m) was selected for the investigation in APR and calcined at 400 °C, to provide a support stable to the reaction conditions (TiO_2_-m400). Its properties were compared with a commercial high-surface area anatase TiO_2_ (TiO_2_-comm). These catalysts were then compared in APR after impregnation of Pt. Both samples were characterized by nitrogen physisorption analysis, powder XRD, ammonia TPD (as bare supports), and TEM (after Pt impregnation). The sample prepared by microemulsion synthesis showed higher surface area even after calcination at 400 °C and smaller pores with a monomodal distribution centered at 4 nm compared to the commercial one (centered at 10 nm) (Figure S5). Moreover, the hysteresis loop is characteristic of a porous material with networks of interconnected pores with increasing size [87,88], as reported in literature for similar microemulsion-synthesized titanium oxides [89].

**Figure 5:**
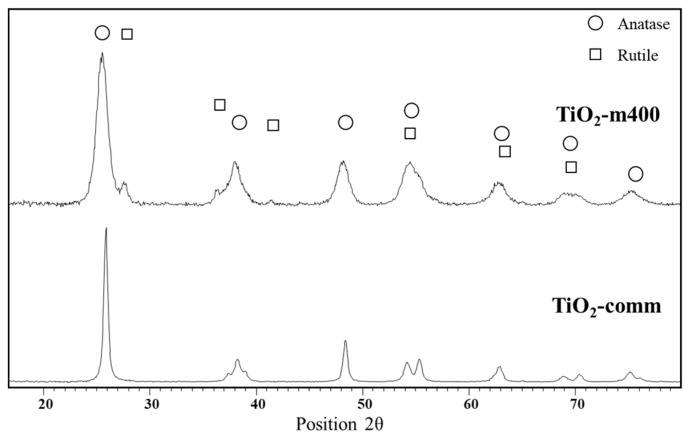
XRD analyses of TiO_2_-m400 and TiO_2_-comm.

On the opposite the commercial sample displayed a porosity ascribable to slit-shaped channels. Powder XRD analyses of TiO_2_-m showed a main anatase phase with 7% of rutile phase while the commercial sample was a pure anatase phase (Figure 5).

**Figure 6:**
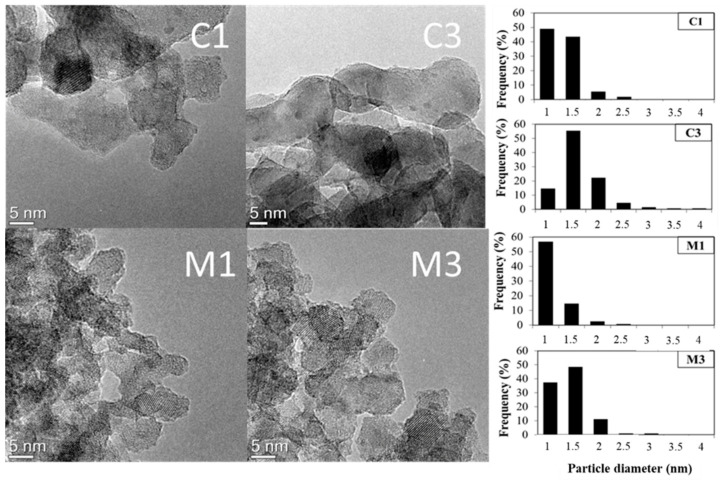
TEM images of different Pt/TiO_2_ samples and related Pt particles distribution: C1 = 1 wt% Pt on TiO_2_-comm; C3 = 3 wt% Pt on TiO_2_-comm; M1 = 1 wt% Pt on TiO_2_-m; C3 = 3 wt% Pt on TiO_2_-m. The dispersion histograms were calculated on 100 Pt nanoparticles.

**Figure 7:**
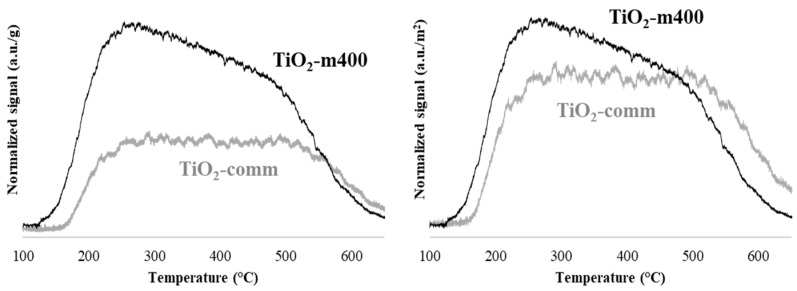
Temperature programmed desorption analysis of NH_3_ (heating from 100 to 650 °C at 10 °C/min, 0.150 g of sample), normalized on mass (**left**) and on surface area (**right**), for TiO_2_-m400 and TiO_2_-comm.

Table 3 reports the dimension of the crystalline domain calculated by Scherrer equation, is reported which was smaller for microemulsion sample. This was also confirmed by TEM which showed well defined spherical-like crystallites for the Pt/TiO_2_-m400 sample while longer and irregular aggregates for Pt/TiO_2_-comm (Figure 6). Surface characterization was deepened analyzing the acid sites of the two titania samples by ammonia temperature programmed desorption. The desorption curves, reported in Figure 7, are centered between 150 and 650 °C, showing for both supports the presence of weak and strong acid sites, with the microemulsion sample having a higher amount of weak acid sites. A comparison within the two samples showed a different total ammonia uptake (Table 3), higher for the microemulsion sample. This demonstrates that a higher density of surface acid sites was obtained thanks to the microemulsion synthesis which provided a high surface area support. To get a further insight in the contribution of the microemulsion synthesis on acid sites, the desorption curve was normalized on the surface area of the sample, thus eliminating the effect of this parameter, which is higher for the microemulsion sample. In this case, a similar total ammonia uptake was observed (Table 3). Nevertheless, the microemulsion sample showed a higher amount of desorbed ammonia at low temperature, compared to TiO_2_-comm, clearly underlying the presence of a higher density of weak acid sites. This may be related to two concurrent factors: weak acid sites derive from the presence of fivefold coordinated Ti^4+^ cations (Ti^4+^_V_), with one unsaturated coordination, while fourfold Ti^4+^ cations with two coordinative unsaturations (Ti^4+^_IV_) are the main responsible for strong acidity [90]. The ordered morphology given to TiO_2_ crystals by the microemulsion synthesis is thought to lead to a homogenous, less uncoordinated surface, with a higher density of Ti^4+^_V_.

### 3.2. Catalytic Activity in APR Reaction

#### 3.2.1. Effect of Reaction Time and Mechanism of Reaction

**Figure 8:**
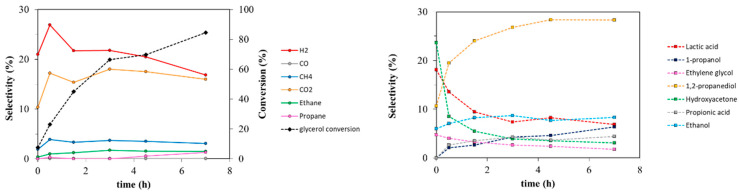
Liquid phase (**right**) and gas phase (**left**) products trend. Reactions performed at 225 °C over 3 wt% Pt/TiO_2_-m400 catalyst; 6 wt% glycerol loading in water.

The catalytic aqueous phase reforming of glycerol was at first studied at 225 °C with 3 wt% Pt/TiO_2_-m400 at different reaction times to evaluate the effect of this parameter on the catalyst performances (Figure 8 and Figure S5). This catalyst was selected as it displayed the highest surface area and an almost pure anatase phase. The rutile-based support was also tested after impregnation with Pt but provided lower hydrogen selectivity, glycerol conversion, liquid product selectivity, and higher carbon loss than the anatase-based one (Figure S6) and was thus excluded from further catalytic investigations.

Glycerol conversion shows a constant increase with reaction time. Hydrogen yield increases concurrently but reaches a plateau over 3 h (Figure S7). H_2_ selectivity was higher at low reaction time, while it decreased with time, indicating hydrogen consumption to give reduced compounds. Hydroxyacetone (HA) and lactic acid (LA) were obtained since the first stages of the reaction showing high selectivity and significant yields at 0 h (just heating up the reactor to 225 °C and immediately cooling it down). HA can be accounted as a primary product. LA is not a primary product but derives from glyceraldehyde and pyruvaldehyde which are unstable under the reaction conditions, as will be discussed later. However, their selectivity decreased at higher reaction times due to the formation of propionic acid (PA) from LA and 1,2-propanediol (1,2-PDO) from HA. Lastly, 1-propanol (PrOH) and propane showed a differently from the products previously reported with a slow, constant increase with reaction time. This suggests that they are products of consecutive reaction from secondary compounds (i.e., 1,2-PDO and 1-PrOH, respectively). CO_2_ production cannot be completely justified by C_2_ (ethanol, ethylene glycol, and ethane) production and may be the sum of decarbonylation, decarboxylation, and in part of reforming and water gas shift processes. As for CH_4_ selectivity, it can result from C–C cleavage to C_2_, combined with a surface recombination with adsorbed hydrogen. Methane is reported to be produced together with EtOH on Ru-based catalysts from 1,2-PDO in H_2_-rich environments [7,91], by prior cleavage of C–O bond and subsequent C–C [92] bond rupture. It can also be given by reforming of methanol (MeOH) or ethanol (EtOH) [93,94] or by CO or CO_2_ methanation [30]. Since CH_4_ selectivity does not vary with time, it may be formed by a complexity of mechanisms: mainly from the production of C_2_, especially from glycerol and ethylene glycol transformation, or by 1,2-PDO and EtOH reforming.

**Figure 9:**
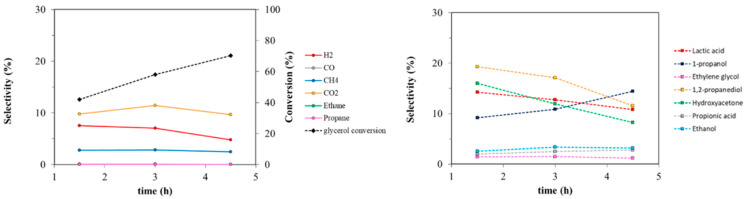
Trend of liquid and gas phase products for tests performed at 250 °C over 1 wt% Pt/TiO_2_-m400. catalyst; 17 wt% glycerol loading in water.

The reaction was also studied at 250 °C with 1 wt% Pt/TiO_2_-m400 considering reaction times of 1.5, 3, and 4.5 h (Figure 9 and Figure S8). Again, glycerol conversion raised, and H_2_ selectivity fell with increasing reaction time, while constant CO and CH_4_ selectivities were detected. Concurrently, HA, 1,2-PDO, LA, and PrOH were found in the liquid phase with high selectivities after 1.5 h, together with a smaller amount of EG. Increasing reaction time led to an increase in PrOH selectivity at the expense of HA, LA, and 1,2-PDO. The obtained trends indicate that LA, 1,2-PDO, and HA are formed in the first stages of reaction, while consecutive products appear to be EtOH, PA, and PrOH. On the other hand, ethylene glycol trend is not still clearly ascribable as primary or secondary product because its selectivity remained constant with time.

**Figure 10:**
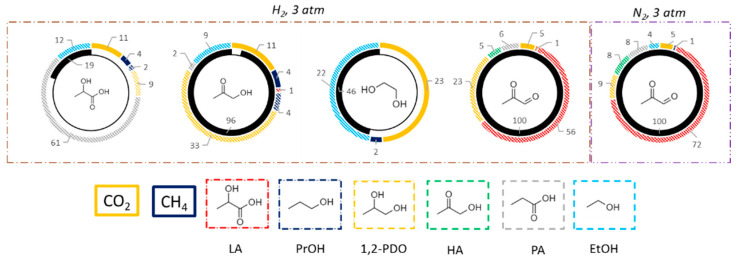
Results of reactivity tests with intermediates in terms of conversion and product selectivity. Tests performed at a temperature of 225 °C for 3 h over 3 wt% Pt/TiO_2_-m400 in water; 3 wt% reagent/water, 0.45 g of catalyst. Inner circle reports conversion (%), outer circle reports selectivities (%).

A further insight in the reaction mechanism was provided performing tests with intermediate of reaction, namely lactic acid, hydroxyacetone, ethylene glycol, and pyruvaldehyde at the same temperature (225 °C) of glycerol APR (Figure 10 and Table S1).

**Figure 11:**
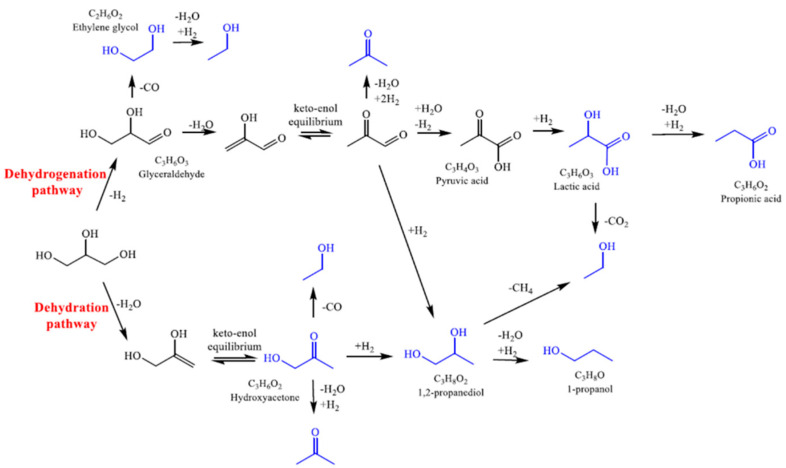
Proposed mechanism of glycerol conversion on Pt/TiO_2_m400. Detected products have been highlighted in blue while black ones are supposed based on literature data.

Reactions were performed under H_2_ atmosphere to simulate the reaction environment the reactions were performed under H_2_ atmosphere (3 bars of hydrogen were loaded at room temperature, before heating the reactor), though pyruvaldehyde was also tested under nitrogen. LA was mainly converted in PA, showing also low selectivity in CH_4_, CO_2_, 1,2-PDO, and EtOH. The latter is produced by decarboxylation of LA together with CO_2_ while methane is obtained by CO_2_ hydrogenation. Direct hydrogenation of LA to PA is the main pathway involved. However, the low LA conversion (19%) indicates that this reagent is quite stable in the reaction conditions employed. HA provides a wider product distribution with high selectivity in CO_2_, EtOH, and 1,2-PDO, with low methane and PrOH formation. EtOH is produced by decarbonylation of HA, while CO_2_ is obtained by WGS reaction of the produced CO. 1,2-PDO is the direct product of HA hydrogenation, while PrOH is given by further dehydration and hydrogenation of 1,2-PDO. Methane can be produced by methanation of CO_2_ or steam reforming of EtOH. When ethylene glycol was reacted in the same conditions, it was selective toward the production of CO_2_ and EtOH. This is consistent with a dehydration/hydrogenation reaction to produce EtOH and the consecutive reforming of the alcohol, or of ethylene glycol itself, followed by WGS to give CO_2_. Finally, pyruvaldehyde reactivity was investigated. This is obtained in the APR process by dehydrogenation and dehydration of glycerol, followed by a keto-enolic tautomerization. However, this intermediate was never detected by HPLC in the other tests of this study, due to its instability and fast reactivity to consecutive products. Pyruvaldehyde can react by two main pathways, namely hydration to pyruvic acid followed by hydrogenation to LA or direct double hydrogenation to 1,2-PDO. Two tests were performed under different atmospheres. If an inert atmosphere is used, which may simulate the first stages of the glycerol APR process, the main product is LA as the first pathway is driven by the presence of water and the absence of hydrogen. In a reductive atmosphere, thus representative of the intermediate stages of the process (where dehydrogenation has already occurred in some extent), the main product from pyruvaldehyde is 1,2-PDO, obtained by double hydrogenation. Thus, analyzing the obtained results and literature data for both APR [30,94,95] and hydrogenolysis reactions [37,88,96], the reaction scheme of Figure 11 was proposed to occur on Pt/TiO_2_-m400.

The reactions occurring over glycerol are dehydrogenation or dehydration pathways. The first is thought to go through glyceraldehyde intermediate. In this way, H_2_ is produced and can further undergo hydrogenation reactions. LA and PA are the main products, with EtOH also possibly produced. In parallel, dehydration of primary C-OH of glycerol can give 1,2-PDO and PrOH by keto-enol equilibrium. The product distribution is mainly influenced though the presence of hydrogen obtained by dehydrogenations favored by Pt, which also favors decarbonylation and C–C cleavage [3,97], and the presence of acid sites which can catalyze the dehydration or hydration reactions.

#### 3.2.2. Comparison within Pt over TiO_2_-m400 and TiO_2_-comm

**Figure 12:**
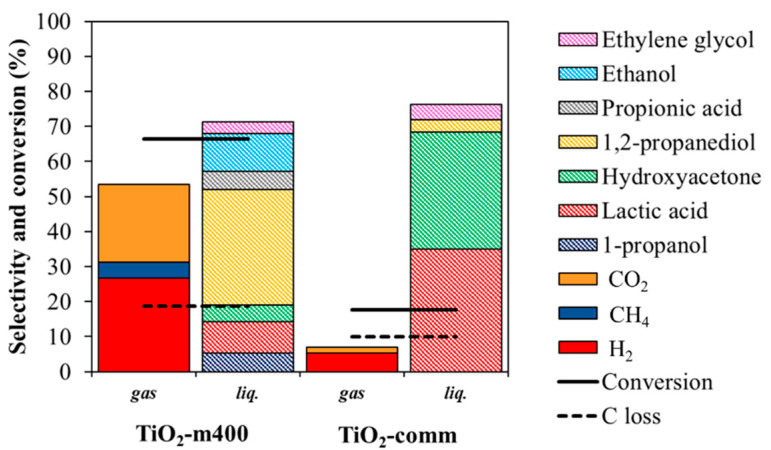
Comparison within 3 wt% Pt loading on TiO_2_-m400 and TiO_2_-comm. Reaction performed at 225 °C for 3 h; 6 wt% glycerol loading in water.

The 3 wt% Pt supported on TiO_2_-m400 catalyst was compared with a commercial support impregnated with the same Pt loading (Figure 12). The differences in glycerol conversion appeared to be significant: in particular, for the microemulsion support, glycerol conversion reached 66% with 27% H_2_, 17% CO_2m_ and 33% 1,2-PDO selectivity at 225 °C and 3 h. CO selectivity was negligible as the produced carbon monoxide was readily consumed by WGS reaction which is favored for both catalysts at the investigated conditions. Moreover, low selectivity in LA, PrOH, HA, EtOH, PA, and EG were observed. The commercial sample provided a 4-times lower glycerol conversion (16%) and major selectivity in products as LA (35%) and HA (33%), with lower selectivity of secondary products (1,2-PDO, PrOH, and CH_4_).

In general, the selectivity to dehydration products (HA, 1,2-PDO, and PrOH) was higher for Pt/TiO_2_-m400 and can be ascribed to the higher surface area of the microemulsion synthetized support, since dehydration reaction occurs over TiO_2_ surface [98]. This was also observed when the tests were carried out on bare supports (Figure S9) suggesting the role of the support in product selectivity. Nevertheless, the absence of a metallic active phase provided low conversions.

MP-AES analysis was carried out on the liquid product solution to assess the presence of solubilized Pt, which was found to be absent indicating that the catalysts were not affected by leaching even under the harsh reaction conditions employed. This can be addressed to the good interaction between Pt and the TiO_2_ support. TEM analysis of the used materials evidenced that Pt nanoparticles with a narrow distribution were still present on the support after the test and that the TiO_2_ material maintained its nano-spherical structure (Figure S10).

**Figure 13:**
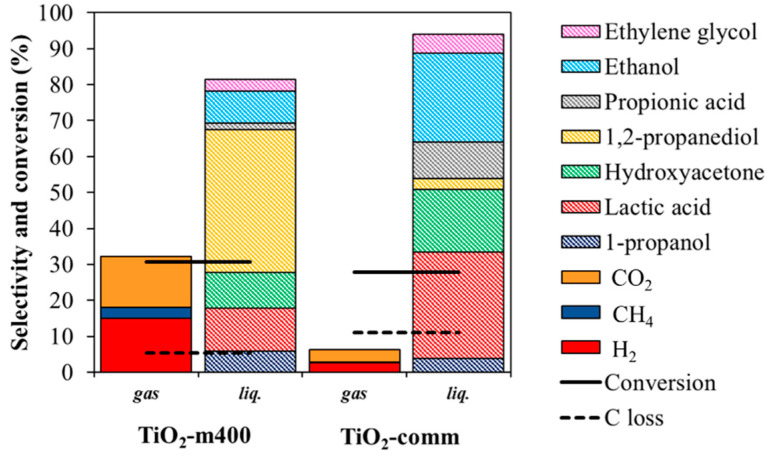
Comparison of the catalytic activity at glycerol isoconversion. 3 wt% Pt loading, time of reaction 3 h, 17 wt% glycerol loading in water. Pt/TiO_2_-m400 test performed at 225 °C, Pt/TiO_2_-comm at 250 °C.

**Figure 14:**
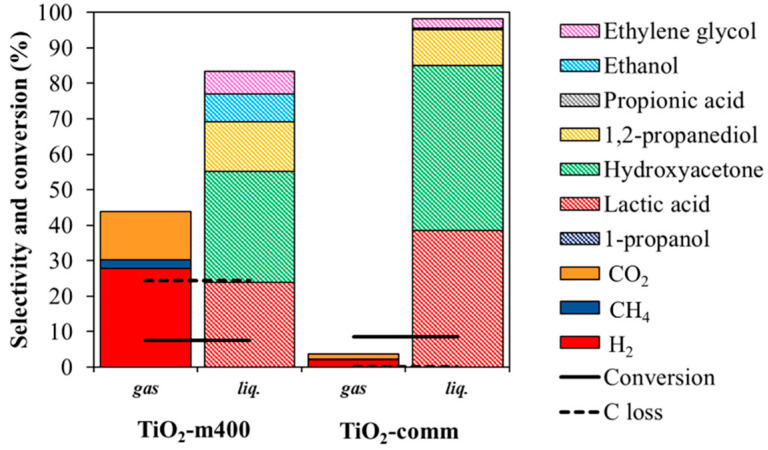
Comparison of the catalytic activity at glycerol isoconversion. 3 wt% Pt loading temperature 225 °C, 6 wt% glycerol loading in water. Pt/TiO_2_-m400 test performed for 0 h, Pt/TiO_2_-comm for 1.5 h.

Moreover, the operative conditions were changed for the two catalyst to obtain similar glycerol conversion, being able to better compare selectivities and understand the reaction pathways of the reaction. At both high and low iso-conversions (Figure 13 and Figure 14 respectively), an enhanced selectivity in gas phase products for the microemulsion sample. Decarbonylation and decarboxylation products and secondary products (1,2-PDO, PrOH, and EtOH) were favored compared to the commercial catalyst, suggesting that the microemulsion sample was able to faster catalyze the reactions involved in the mechanism depicted in Figure 11.

The differences in activity and in product selectivity could be explained by considering metal-support cooperation, as the two catalysts had similar Pt particle size and distribution. It is reported in literature that the adsorption of an alcohol to form an alkoxy over both TiO_2_ and metal is followed by a different reactivity [98]. Over TiO_2_, the alkoxy undergoes mono or bimolecular dehydration to alkenes or ethers respectively, while over a metal site decarbonylation or dehydrogenation are favored. It is also suggested a rapid exchange within TiO_2_ adsorbed species and adjacent metallic particles. As a larger number of acidic sites are present on the microemulsion synthetized titania, a larger adsorption of species on the oxide is supposed to occur with a fast exchange of intermediates from the support to the metal, favored by the larger presence of weak acid sites. This enhanced the conversion of support-adsorbed species and provided higher activity for Pt/TiO_2_-m400 compared to the commercial supported catalyst.

#### 3.2.3. Effect of Metal Loading

**Figure 15:**
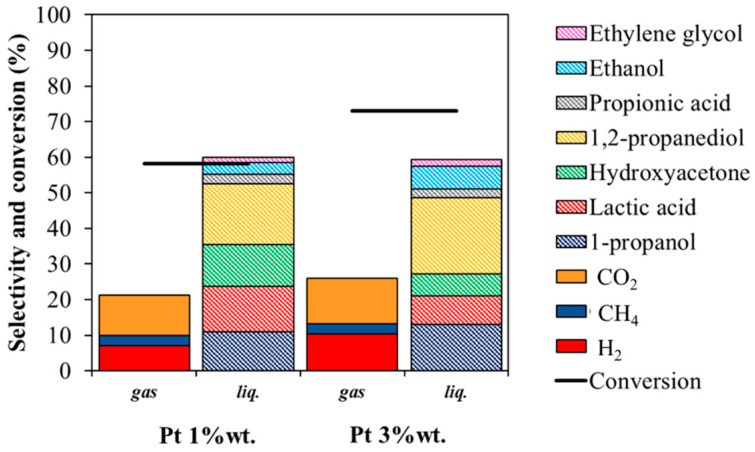
Gas and liquid selectivity and conversion for 1% and 3 wt% Pt/TiO_2_-m400. Reactions performed at 250 °C for 3 h; 17 wt% glycerol in water.

To get a better insight on the role of Pt loading both 1 wt% Pt and 3 wt% Pt impregnated on TiO_2_-m400 support were tested at 250 °C for 3 h (Figure 15). Increasing the metal content provided higher conversion, while selectivity in hydrogen slightly increased. Although some changes in liquid product selectivity was observed, the selectivity sums of dehydrogenation pathway products (LA, EtOH, EG, and PA; 27% for Pt1% and 25% for Pt3%) and dehydration pathway products (HA, 1,2-PDO, and PrOH; 53% for Pt1% and 54% for Pt3%) remained approximately constant. That suggests that increasing the metal loading did not influence the overall glycerol activation but only fostered the reaction rate and thus the amount of consecutive products.

## 4. Conclusions

The optimization of a microemulsion technique for the synthesis of TiO_2_ wias performed, which allowed to obtain small and regular TiO_2_ nano spheres characterized by high surface area even after calcination at 400 °C. The homogeneous morphology of the TiO_2_ nanospheres, obtained thanks to the peculiarity of the microemulsion synthesis, where micelles acted as confined reaction environment provided a higher density of weak acid sites, which, together with the higher surface area, resulted in higher total acidity compared to commercial TiO_2_. The homogeneous morphology is supposed to provide a high density of fivefold coordinated Ti^4+^ cations (Ti^4+^_V_), with one unsaturated coordination, which are responsible for weak acidity. The synthetized and commercial TiO_2_ supports were loaded with Pt and tested in APR reaction of glycerol for hydrogen production. The study of obtained products and reaction intermediates allowed to propose a reaction mechanism evidencing that the main products derive from both dehydration and dehydrogenation pathways. Moreover, the comparison of the catalytic activity of the synthesized material with the commercial support evidenced the superior performances of the former in terms of both activity and hydrogen selectivity. Moreover, a higher selectivity toward secondary products (1,2-propanediol, 1-propanol, propionic acid, and ethanol) was observed for the microemulsion sample and was connected with the higher acidity of the microemulsion sample. Considering that the Pt particle dimensions and Pt loading were similar, the large difference in conversion and selectivity was due to an enhanced cooperation between the acid sites of nanocrystals TiO_2_ obtained by microemulsion and the redox sites of the Pt particles deposited on the TiO_2_ surface. This cooperation was favored by the higher surface area and density of acid sites given by the microemulsion synthesis, linked with high Pt dispersion in the form of nanometric particles. Future studies will focus on increasing the acid properties of TiO_2_-based materials and their interaction with the active phase, which would further increase hydrogen selectivity, diving the reaction toward the formation of consecutive products.

## Figures and Tables

**Table 2:** Characterization data of as-synthetized, uncalcined TiO_2_ samples obtained with different synthetic methods. * Specific surface area obtained by Brunauer-Emmett-Teller (BET) analysis.

Sample	Surf/Co-Surf.	Stirring Time at r.t.	Heating Type	Heating Time (h)	Rutile vs. Anatase Phase (%)	Particle Dimension Anatase (nm)	SSA * (m^2^/g)
TiO_2_-m	Yes	1 h	Reflux	5	7:93	4	319
TiO_2_-e	No	1 h	Reflux	5	64:36	5	199
TiO_2_-5 days	Yes	5 days	-	-	91:9	9	123
TiO_2_-120 °C	Yes	1 h	Autoclave (120 °C)	4	1:99	5	249

**Table 3:** Characterization of TiO_2_-m400 and TiO_2_ commercial sample.

	Crystal Phase	Particle Size (nm)	SBET (m^2^/g) ^1^	Pore Avg. Diameter (nm)	Vpore (cm^3^/g)	NH_3_ Uptake (mmol/g)	NH_3_ Uptake (mmol/m^2^)
TiO_2_-m400	Anatase	8	147	4.2	0.15	18.94	0.13
TiO_2_-comm	Anatase	24	90	14.6	0.32	10.35	0.12

^1^ Specific surface area obtained by BET analysis.

## Data Availability

Not applicable.

## Supplementary Materials

The following are available online at https://www.mdpi.com/article/10.3390/nano11051175/s1, Figure S1: SEM images of the sponge-like solid deposited on the bottom of the flask soon after the synthesis, Figure S2: XRD analysis of TiO_2_ samples synthesized with (TiO_2_-m) and without (TiO_2_-e) the presence of a surfactant and co-surfactant, Figure S3: XRD analyses of TiO_2_ samples synthesized with different synthetic methods, Figure S4: Schematic representation of the kinetically controlled and thermodynamically controlled crystal growth leading to anatase or rutile; each octahedra represent a TiO_6_^2−^ anion, Figure S5: Adsorption/desorption isotherms and pore distribution of the TiO_2_ samples, Figure S6: Comparison of APR reactivity within Pt 3 wt% on TiO_2_-m400 and Pt 3 wt% on TiO_2_-5 days400. Reaction performed at 225 °C for 3 h; glycerol loading 6 wt%. Figure S7: Liquid phase (centre), gas phase (left) products yields and conversion and carbon loss (right). Reactions performed at 225 °C over 3 wt% Pt/TiO_2_-m400 catalyst; 6 wt% glycerol loading in water. Figure S8: Liquid phase (centre), gas phase (left) products yields and conversion and carbon loss (right). Reactions performed at 250 °C over 1 wt% Pt/TiO_2_-m400 catalyst; 17 wt% glycerol loading in water. Figure S9: Comparison within TiO_2_-m and commercial supports without Pt impregnation. Reaction performed at 250 °C for 3 h; glycerol loading 17%. Figure S10: TEM analysis of Pt 3 wt% on TiO_2_-m400 after the aqueous phase reforming reaction performed at 225 °C for 3 h; 6 wt% glycerol loading in water. Arrows indicate Pt nanoparticles. Table S1: Results of reactivity tests with intermediates in terms of conversion and product selectivity. Reactions performed at 225 °C for 3 h over 3 wt% Pt/TiO_2_-m400 in water; 3 wt% loading of reagent in water, 0.45 g of catalyst. Inner circle reports conversion (%), outer circle reports selectivities (%). Table S2: Glycerol conversion, hydrogen selectivity and liquid product selectivity for some Pt based catalysts reported in literature.
