# Supplementary material for: Microemulsion Derived Titania Nanospheres: An Improved Pt Supported Catalyst for Glycerol Aqueous Phase Reforming
Source: Nanomaterials (Basel). 2021 Apr 29;11(5):1175. doi: 10.3390/nano11051175 (PMC8144991; doi:10.3390/nano11051175)
Supplement: Supplementary file 1 [file nanomaterials-11-01175-s001.zip › nanomaterials-1174944-supplementary.pdf]

Supplementary Materials

Microemulsion Derived Titania Nanospheres: An Improved Pt Supported Catalyst for Glycerol Aqueous Phase Reforming

Andrea Fasolini, Erica Lombardi, Tommaso Tabanelli and Francesco Basile *

Dipartimento di Chimica Industriale "Toso Montanari", Alma Mater Studiorum-Università di Bologna, Viale del Risorgimento 4, 40136 Bologna, Italy; andrea.fasolini2@unibo.it (A.F.); erica.lombardi3@gmail.com (E.L.); tommaso.tabanelli@unibo.it (T.T.)
* Correspondence: f.basile@unibo.it; Tel.: +39-051-2093663

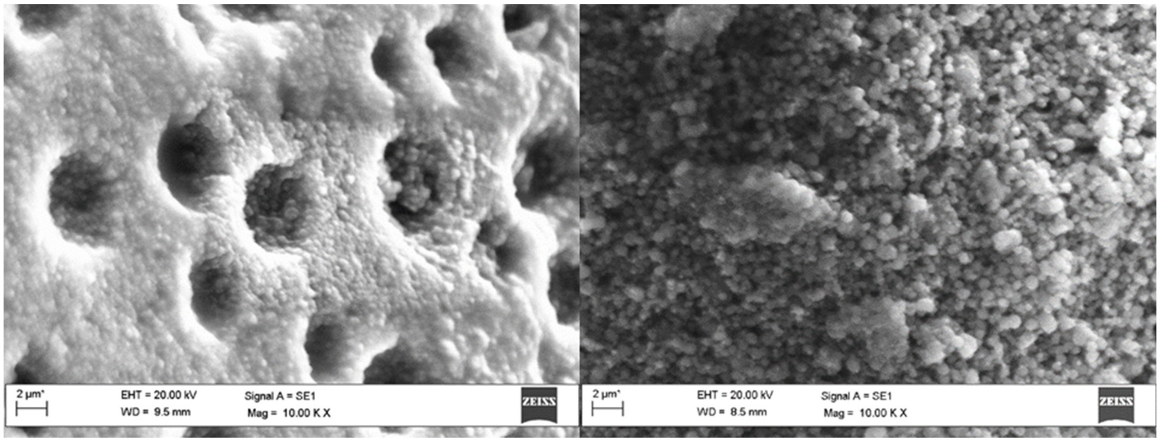

Figure S1. Scanning electron microscopy (SEM) images of the sponge-like solid deposited on the bottom of the flask soon after the synthesis.

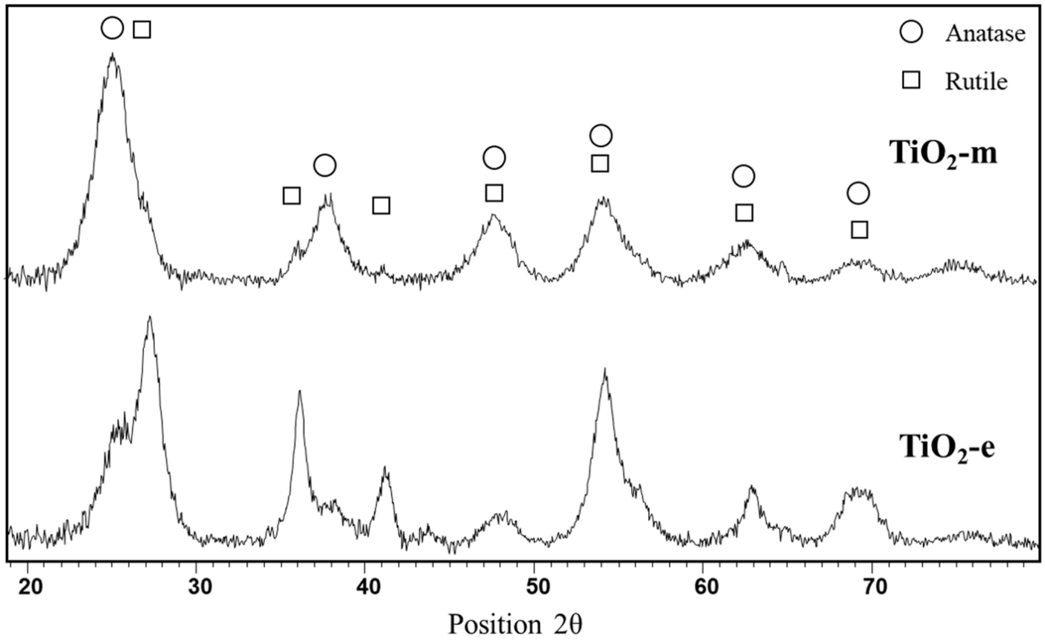

Figure S2. X-ray diffraction (XRD) analysis of TiO_2 samples synthesized with (TiO_2-m) and without (TiO_2-e) the presence of a surfactant and co-surfactant.

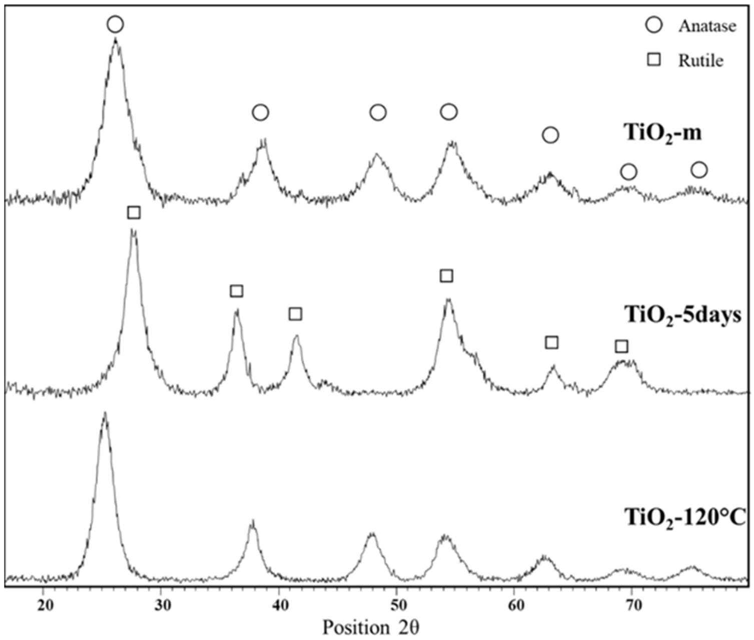

Figure S3. XRD analyses of TiO_2 samples synthesized with different synthetic methods (see Table 1).

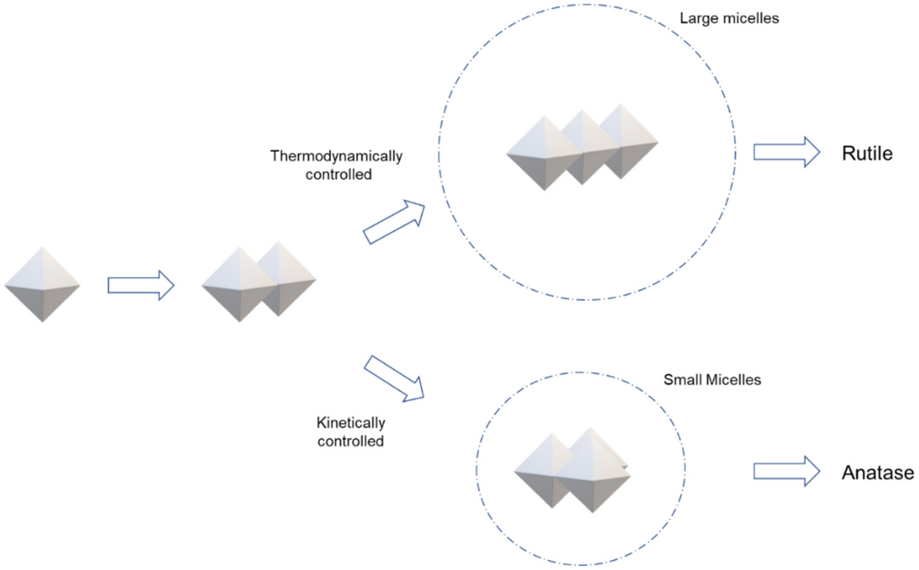

Figure S4. Schematic representation of the kinetically controlled and thermodynamically controlled crystal growth leading to anatase or rutile; each octahedra represent a TiO_6^{2-} anion.

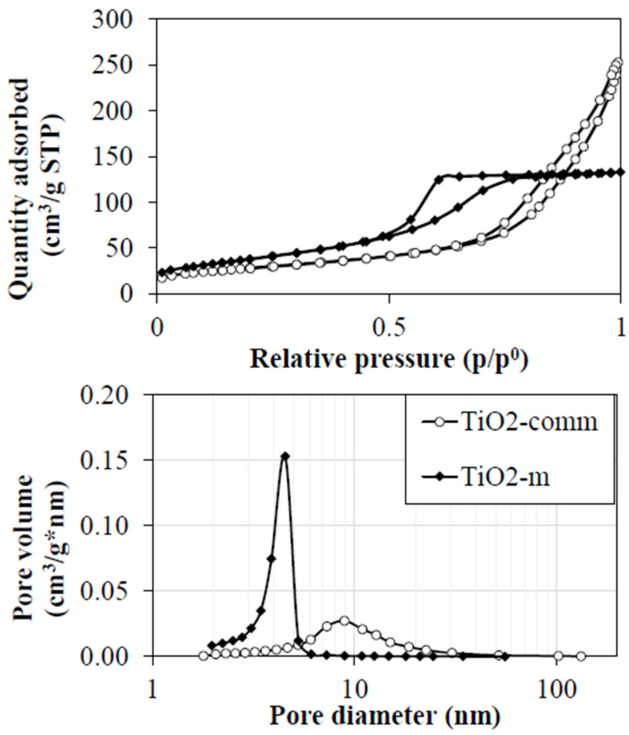

Figure S5. Adsorption/desorption isotherms and pore distribution of the TiO_2 samples.

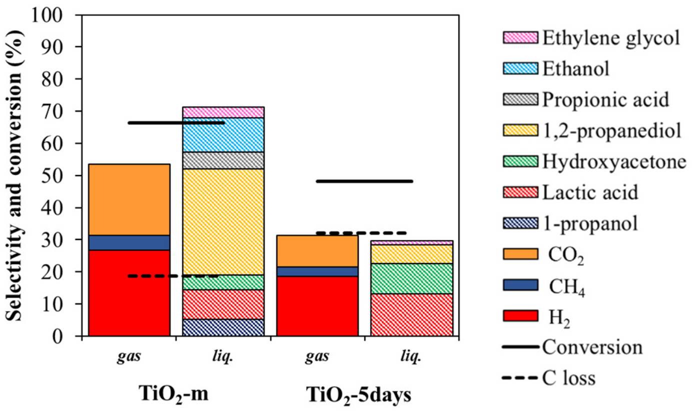

Figure S6. Comparison of glycerol aqueous phase reforming (APR) reactivity within Pt 3 wt% on TiO_2-m400 and Pt 3 wt% on TiO_2-5days400. Reaction performed at 225 °C for 3 h; glycerol loading 6 wt%.

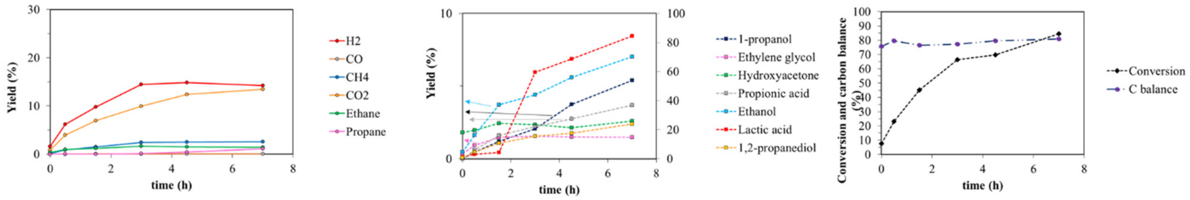

Figure S7. Liquid phase (**centre**), gas phase (**left**) products yields and conversion and carbon loss (**right**). Reactions performed at 225 °C over 3 wt% Pt/TiO_2-m400 catalyst; 6 wt% glycerol loading in water.

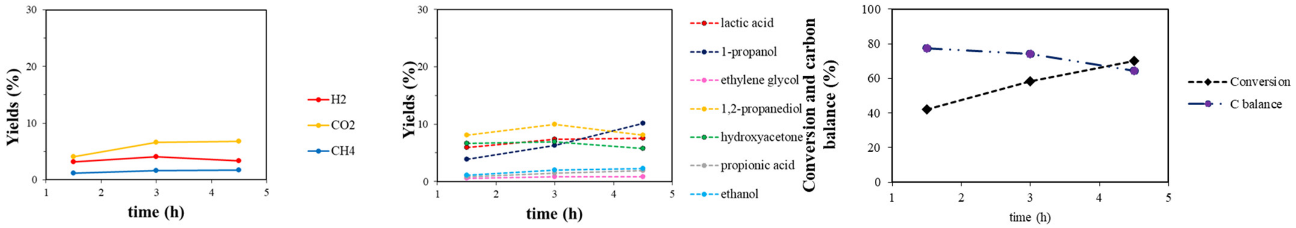

Figure S8. Liquid phase (**centre**), gas phase (**left**) products yields and conversion and carbon loss (**right**). Reactions performed at 250 °C over 1 wt% Pt/TiO_2-m400 catalyst; 17 wt% glycerol loading in water.

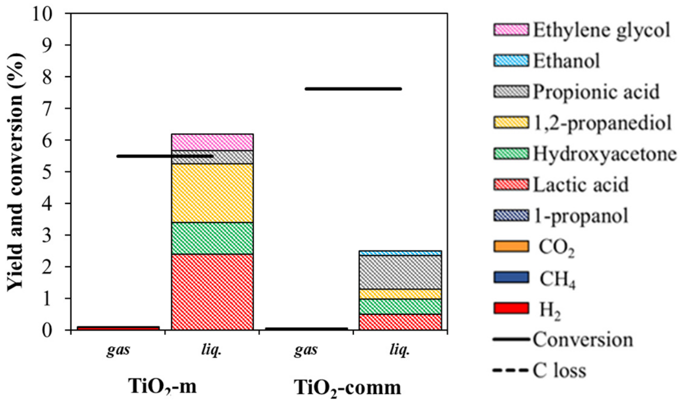

Figure S9. Comparison within TiO_2-m and commercial supports without Pt impregnation. Reaction performed at 250 °C for 3 h; glycerol loading 17 wt%.

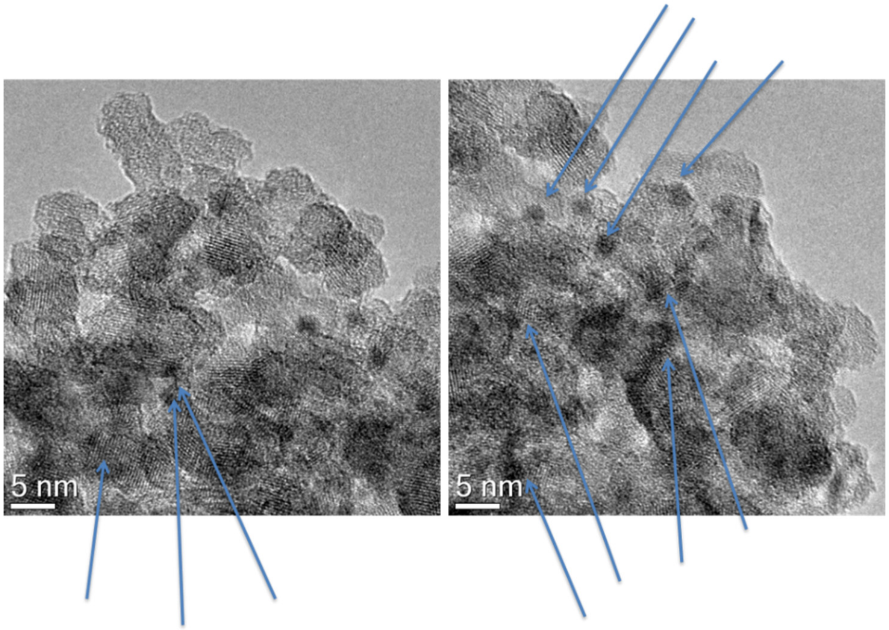

Figure S10. TEM analysis of Pt 3% wt on TiO_2-m400 after the aqueous phase reforming reaction performed at 225 °C for 3 h; 6 wt% glycerol loading in water. Arrows indicate Pt nanoparticles.

Table S1. Results of reactivity tests with intermediates in terms of conversion and product selectivity. Reactions performed at 225 °C for 3 h over 3 wt% Pt/TiO_2-m400 in water; 3 wt% loading of reagent in water, 0.45 g of catalyst. Inner circle reports conversion (%), outer circle reports selectivities (%).

Reagent	P (H_2) bar	Conv	CO_2	CH_4	LA	PrOH	1,2-PDO	HA	PA	EtOH
LA	3	19	11	4	-	2	9	0	61	12
HA	3	96	11	4	1	4	33	-	2	9
EG	3	46	23	2	0	0	0	0	0	22
Pyruvaldehyde	-	100	5	1	72	0	9	8	8	4
Pyruvaldehyde	3	100	5	1	56	0	23	5	6	0

Table S2. Glycerol conversion, hydrogen selectivity and liquid product selectivity for some Pt based catalysts reported in literature. * Results reported as hydrogen yield.

Catalyst	T (°C)	Time (h)	Process	Conv. (%)	Sel. H_2	Sel. 1,2-PDO	Sel. LA	Sel. HA	Sel. EG	Sel. PrOH	Sel. PA	Sel. EtOH	Ref.
Pt/TiO_2	225	/	Fixed Bed	27	56*	n.a.	n.a.	n.a.	n.a.	n.a.	n.a.	n.a.	[1]
Pt/TiO2	210	6	Batch	46	17*	49	n.a.	n.a.	12	2	n.a.	10	[2]
Pt/Al_2O_3	250	/	Fixed bed	43	85	n.a.	n.a.	n.a.	n.a.	n.a.	n.a.	n.a.	[3]
Pt/MgO	225	/	Fixed bed	n.a.	28	n.a.	n.a.	n.a.	n.a.	n.a.	n.a.	n.a.	[1]
Ru/Al_2O_3 + Pt/Al_2O_3	220	6	Batch	50	n.a.	47	2	n.a.	6	n.a.	n.a.	1	[4]
Pt-Ir-ReO_x/SiO_2	190	17	Batch	30	n.a.	19	n.a.	n.a.	1	4	2	2	[5]
Pt/Al_2O_3	210	6	Batch	10	31*	21	n.a.	n.a.	32	n.a.	n.a.	6	[2]

References

1. Guo, Y.; Azmat, M.U.; Liu, X.; Wang, Y.; Lu, G. Effect of Support's Basic Properties on Hydrogen Production in Aqueous-Phase Reforming of Glycerol and Correlation between WGS and APR. *Appl. Energy* **2012**, *92*, 218–223, doi:10.1016/j.apenergy.2011.10.020.
2. Delgado, S.N.; Yap, D.; Vivier, L.; Especel, C. Influence of the Nature of the Support on the Catalytic Properties of Pt-Based Catalysts for Hydrogenolysis of Glycerol. *J. Mol. Catal. Chem.* **2013**, *367*, 89–98, doi:10.1016/j.molcata.2012.11.001.
3. Lehnert, K.; Claus, P. Influence of Pt Particle Size and Support Type on the Aqueous-Phase Reforming of Glycerol. *Catal. Commun.* **2008**, *9*, 2543–2546, doi:10.1016/j.catcom.2008.07.002.
4. Roy, D.; Subramaniam, B.; Chaudhari, R.V. Aqueous Phase Hydrogenolysis of Glycerol to 1,2-Propanediol without External Hydrogen Addition. *Catal. Today* **2010**, *156*, 31–37, doi:10.1016/j.cattod.2010.01.007.
5. Liu, S.; Tamura, M.; Shen, Z.; Zhang, Y.; Nakagawa, Y.; Tomishige, K. Hydrogenolysis of Glycerol with In-Situ Produced H_2 by Aqueous-Phase Reforming of Glycerol Using Pt-Modified Ir-ReOx/SiO_2 Catalyst. *Catal. Today* **2018**, *303*, 106–116, doi:10.1016/j.cattod.2017.07.025.
